# Spheroid arrays for high-throughput single-cell analysis of spatial patterns and biomarker expression in 3D

**DOI:** 10.1038/srep41160

**Published:** 2017-01-30

**Authors:** Delyan P. Ivanov, Anna M. Grabowska

**Affiliations:** 1 Cancer Biology, Division of Cancer and Stem Cells, School of Medicine, Queen’s Medical Centre, University of Nottingham, Nottingham, UK

## Abstract

We describe and share a device, methodology and image analysis algorithms, which allow up to 66 spheroids to be arranged into a gel-based array directly from a culture plate for downstream processing and analysis. Compared to processing individual samples, the technique uses 11-fold less reagents, saves time and enables automated imaging. To illustrate the power of the technology, we showcase applications of the methodology for investigating 3D spheroid morphology and marker expression and for *in vitro* safety and efficacy screens. First, spheroid arrays of 11 cell-lines were rapidly assessed for differences in spheroid morphology. Second, highly-positive (SOX-2), moderately-positive (Ki-67) and weakly-positive (βIII-tubulin) protein targets were detected and quantified. Third, the arrays enabled screening of ten media compositions for inducing differentiation in human neurospheres. Last, the application of spheroid microarrays for spheroid-based drug screens was demonstrated by quantifying the dose-dependent drop in proliferation and increase in differentiation in etoposide-treated neurospheres.

Current preclinical studies for drugs and safety assessments for chemicals use *in vitro* cell cultures and animal data to predict human response. *In vitro* data generated in 2D models are notoriously unreliable due to the reductionist way of culturing cells as a monolayer on plastic, and animal studies often fail to predict how drugs will behave in humans due to substantial interspecies differences^1^.

Three-dimensional *in vitro* spheroid and organoid models are considered to be more physiologically relevant models of normal and diseased human tissues compared to cells cultured in 2D^2^. Although three-dimensional cultures offer more realistic cell-cell and cell-matrix interactions, there have been two main obstacles for their adoption in drug screening. First, the methods for their culture were low-throughput and resulted in large variation in spheroid size. This has recently been overcome with the introduction of high-throughput plate-based platforms for 3D culture. Second, the techniques to analyze spheroid viability, morphology, gene and protein expression were also slow and laborious. Here, we present a device which overcomes this issue by allowing users to arrange up to 66 spheroids in a single plane for high-throughput downstream analysis of three-dimensional cell cultures.

Three-dimensional aggregate cultures were first described in the 1950s by Moscona^3^, and the advantages of using spheroids in cancer research were recognized in the 1970s by Sutherland^4^. The introduction of plate-based platforms for spheroid culture in hanging-drop^5^,^6^ or liquid overlay^7^,^8^ has enabled researchers to produce a single-spheroid per well and control spheroid size in a high-throughput format. The increased adoption of spheroid screens has mostly relied on plate-based viability measurements^9^,^10^,^11^,^12^. While cytotoxicity assays may be useful in assessing the effectiveness of anticancer drugs, they do not provide clues on the mechanisms behind tumor drug resistance and the adverse outcome pathways leading to toxicity in normal tissues^13^.

The next research frontier is to move away from simplistic viability assays and analyze spheroid morphology and biological function at the single cell level within their 3D context. Spheroid morphology and single cell protein/gene analysis can identify spatial patterns in expression due to nutrient and oxygen gradients and the phenotype of small populations of cells resistant to drug therapy. These properties can be examined using histological and immunohistochemistry techniques. To this end, many technical replicate spheroids are cultured under the same conditions, fixed, embedded in matrix (e.g. agarose gel), frozen or paraffin-embedded, then sectioned, stained and imaged^7^,^12^.

When more than two conditions are examined (e.g. compound screens, dose-response curves) or if many different cell types are used, the replicates from each condition need to be embedded as separate samples (Fig. 1a). In this way a single dose-response assay in a 96-well plate with nine drug levels and one untreated control would yield 10 separate samples per plate and would require at least 30 (3 per condition) microscope slides per protein(Fig. 1a, bottom panel). The increase in number of samples means researchers waste more time to section and stain the samples and use greater amounts of expensive reagents, such as antibodies. Moreover, the random distribution of spheroids in the embedding media necessitates manual imaging, further increasing the time for analysis. The whole process becomes very low-throughput, and requires a big investment in researcher hands-on time and reagents.

We have developed and share the designs of a device which creates spheroid microarrays by transferring previously-fixed spheroids from the cell culture plate to a precast agarose mold (Fig. 1b). The mold-making device (Mold-maker) produces agarose molds with 66 holes arranged in 6 rows and 11 columns. The holes have a paraboloid shape and equal depth and serve to arrange up to 66 spheroids in the same plane. Embedding all spheroids in a common plane reduces the chances for sample mix-up and allows researchers to cut sections containing all 66 spheroids at the same time for simultaneous staining and analysis. Compared to embedding technical replicates in separate blocks, the techniques uses 11 times less reagents and consumables (Fig. 1a, top panel). In contrast to randomly-arranged spheroids in each block, the common embedding plane allows for automated scanning of all spheroids and automated analysis. Spheroid tissues are preserved in the pre-arranged format and can be stored for years at room temperature. This will enable the creation of *in vitro* cell culture libraries directly comparable to patient tissue and xenografts. We also share two open-source macros (Supplementary macros 1 and 2) for the Fiji distribution of ImageJ^14^ which determine either the relative positively stained area or number of nuclei respectively for diaminobenzidine/hematoxylin based immunohistochemistry staining.

This technique will be useful to scientists working on *in vitro* models of cancer, regenerative medicine, stem cells and toxicology. The device can be utilized to compare the morphological and expression profiles of different cell-lines and primary cells. In experimental setups testing a single cell type, the devices can be used to analyze how culture size, media components, growth factors or drugs affect morphology and marker expression.

## Results

### Making spheroid arrays

**Figure 2:**
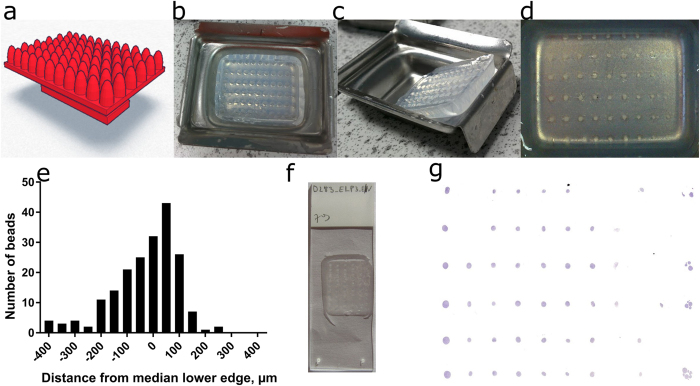
The Mold-maker device, the agarose molds it produces and the final spheroid arrays. (**a**) Three-dimensional plot of the Mold-maker device. It is made up of 66 paraboloid-shaped pegs on a single plane and features a handle for easy removal from the gel. (**b**) Agarose gel after Mold-maker has been removed. (**c**) Side view of the agarose gel. (**d**) Spheroids from 11 cell-lines embedded in a single agarose array. (**e**) Histogram of the average distance from the median lower edge for r = 500 μm beads from three independent experiments. Separate histograms and images are available in the Supplementary information. (**f**) Paraffin section of the spheroid microarray. (**g**) Scan of a representative spheroid array, conditions of interest are located in columns 1–10, column 11 contains multiple marking spheroids.

The Mold-maker is a plastic device which floats on top of hot (50–70 °C) agarose solution to form an agarose mold with 66 equal-depth replicate cavities. The design evolved through a series of prototypes created on a 3D printer for rapid prototyping (Supplementary design 1). The layout of the mold matches the organization of a 96-well plate in which the outer wells are filled with water to prevent evaporation edge-effects. The final design (Fig. 2a) features paraboloid shaped pegs to facilitate pipetting from the top and center spheroids at the bottom of the resultant agarose mold (Fig. 2b–d).

To quantify whether all spheres in the agarose molds lie at a common plane, we embedded plastic beads (r = 500 μm) in the recipient molds and cross-sectioned them along each row. The common plane was defined as the median lower edge for the particles in each row and the distance from this plane plotted on a frequency plot (Fig. 2e). The interquartile range of embedding from the median plane was −100 μm to 50 μm, whereas the 10^th^ and 90^th^ percentiles were −200 μm to 100 μm, for 197 spheres, embedded in three separate molds. This indicates that most spheroids will appear in the same sections provided that their diameter is at least 300 μm. Larger spheroids (500 μm–1 mm) are easier to see with the naked eye. They cover the range most commonly used in spheroid cultures^9^, are very easy to position in the arrays and can yield 50–100 useful histological sections. In illustration, for 500 μm sized spheroids, the theoretical number of 4 μm sections within 20% of the spheroid center above and below the midline is 50 sections. Apart from lacking the physiologically-relevant gradients in nutrients and oxygen, spheroids smaller than 200 μm in diameter are difficult to see and would require a different setup to section in high-throughput.

The agarose molds were fit for the purpose of creating spheroid tissue microarrays. We embedded multicellular spheroids in the arrays (Fig. 2d), sectioned the paraffin-embedded blocks (Fig. 2f), stained the sections with hematoxylin and eosin (H&E) and scanned them (Fig. 2g). We advocate the use of at least 6 replicates per condition as spheroids can be lost during feeding or transfer. While some replicates appeared only in early sections and others in the final ones, there were around 50 sections in the spheroid microarray block which contained the majority of spheroids (Fig. 2g). Spheroid-filled spots can be distinguished on wet paraffin sections during sectioning as opaque dots on the semi-transparent agarose when held against the light. The position of the spheroids can be ascertained by using markers wells which contain large spheroids (Fig. 2g first row), multiple spheroids (Fig. 2g last row) or marker spots^15^. The method has now been tested by 7 researchers (MSc and PhD students and post-doctoral fellows) from different laboratories in the University of Nottingham. While it can take novice researchers up to an hour to prepare an array with 66 spheroids, experienced scientists can produce the arrays twice as fast at an average of 30 minutes per array. Small (300–400 μm) spheroids are harder to see and take longer to transfer from the culture plate to the agarose array, whereas larger (500–1000 μm) spheroids can be transferred in less than 5 minutes.

### Application in assessment of morphology

**Figure 3:**
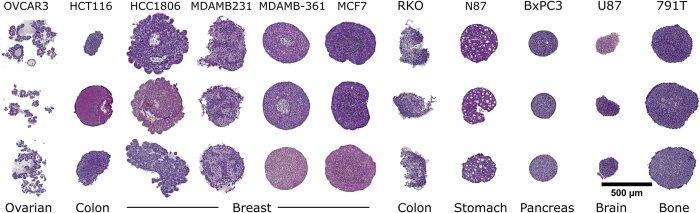
Hematoxylin and eosin staining of multi-spheroid arrays made up of different cell-lines. Each column represents a different cell line, while rows are made up of replicate spheroids. OVCAR-3, HCC1806, MDA-MB-231, MCF-7, N87 were seeded at 16,000 cells/well, while RKO, BxPC3s, HCT116, U87 and 791T at 8,000 cells/well. All spheroids were cultured for 4 days as specified in the Methods section. Scale bar 500 μm.

Spheroid microarrays can be used to rapidly screen 3D cultures from multiple cell-lines for morphology differences. We created spheroid microarrays from 11 different cell-lines derived from 7 tissues (Fig. 3). Spheroid morphology was different for each cell-line showing utility in cell line authentication and phenotype validation. OVCAR3 formed loose aggregates arranged in structures resembling poorly differentiated follicles. The two colon cell-lines formed spheroid with vastly different morphology. The HCT116 cell line did not require BME addition and formed compact spheroids. In contrast RKO cells formed spheroids only after adding BME (250 μg/mL) and the cells in the resulting spheroids were aggregating loosely. The panel of four breast cancer cell-lines showed necrotic cores and complex morphology. HCC1806 had the most complex shape with multiple projections coming out of the spheroid. MDA-MB-231 formed a heterogeneous spheroid with mesenchymal appearance. MDA-MD-361 and MCF-7 formed spheroids with a faintly or strongly staining core respectively which point to the development of hypoxia or necrosis. N87 stomach cancer cells showed parenchymal-like morphology consistent with their origin from a liver metastasis. BxPC3 cells formed very dense cultures and their shape was closest to a perfect sphere compared to the other cell-lines. Both U87 glioma and 791T osteosarcoma cell-lines formed slightly irregular dense spheroids.

### Application in assessment of biomarker expression

**Figure 4:**
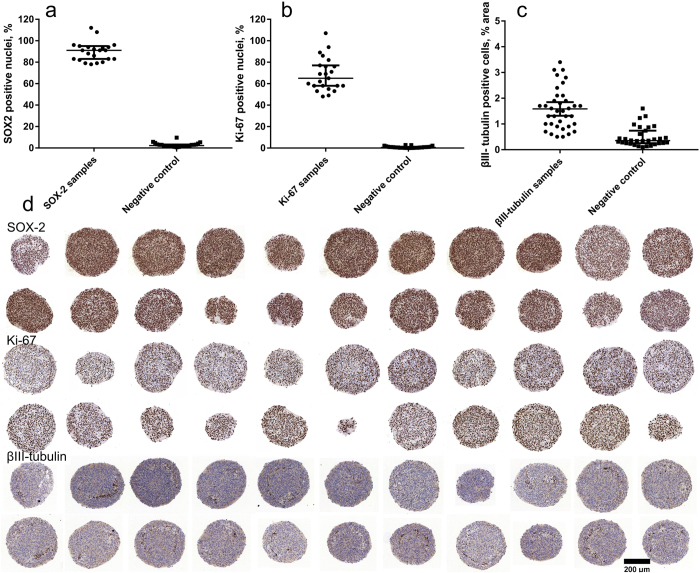
Signal uniformity assessment for antibody-stained protein targets in spheroid microarrays of human fetal brain neurospheres (day 3 of culture). (**a**) Percentage of nuclei positive for SOX-2 (high signal). (**b**) Percentage of nuclei positive for Ki-67(moderate signal), (**c**) Percentage of area positive for βIII-tubulin (low signal.) Signals for the stained samples (circles) are compared to negative isotype controls (squares). (**d**) Scanned images of a selection of the spheroid replicates used in the analysis. First two rows stained with SOX-2, third and fourth (Ki-67), fifth and sixth (βIII-tubulin). Blue-hematoxylin nuclear stain, brown-DAB positive stain. Scale bar 200 μm. In graphs a-c, the line represents the median, the error bars the interquartile range and the dots are individual values for the scoring. Note the difference in scale for the y-axis in panel c.

Antibody-based staining of protein targets was assessed in human fetal brain neurospheres cultured in neural stem cell media. The spheroids contained a high proportion of progenitors (SOX-2), moderate number of proliferating cells (Ki-67) and a low percentage of neurons (βIII-tubulin). To demonstrate signal uniformity the neurospheres were stained for these markers by immunohistochemistry. For the high signal, the mean percentage of SOX-2 positive cells (Fig. 4a) in the neurospheres was 91% (SD = 11, n = 23 spheroids). The moderately-expressed proliferation marker Ki-67 (Fig. 4b) was expressed in 68% of cell nuclei (SD = 15, n = 23). The area of βIII-tubulin positive cells (Fig. 4c) was 1.6% (SD = 0.8, n = 37). Neuronal differentiation was low in the NSC media cultured neurospheres, confined to a nexus in the spheroid (Fig. 4d). A network-like clustering of βIII-tubulin-positive cells was revealed in the area half-way between the neurosphere periphery and the core (Fig. 4d). The data showed a number of outliers due to the different spatial distribution of positive cells within the sample spheroids and imaging artifacts (e.g. hematoxylin aggregates) among the negative controls. We employed the robust version of the Z-factor^16^ based on the median value and the median absolute deviation (MAD) as an outlier-insensitive measure of the separation between the signals for the sample and negative control as shown in equation (1):


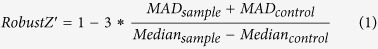


Employing the common acceptance criteria of Z′ > 0.5, the robust Z′ was excellent for SOX-2 and Ki-67 (0.80 and 0.54 respectively) and showed unfavorable overlap for βIII-tubulin (Z′ = 0). That means that treatments which decrease SOX-2 and Ki-67 would be readily quantifiable under these screening conditions, while treatments which decrease the number of neurons would not.

### Use in monitoring differentiation

**Figure 5:**
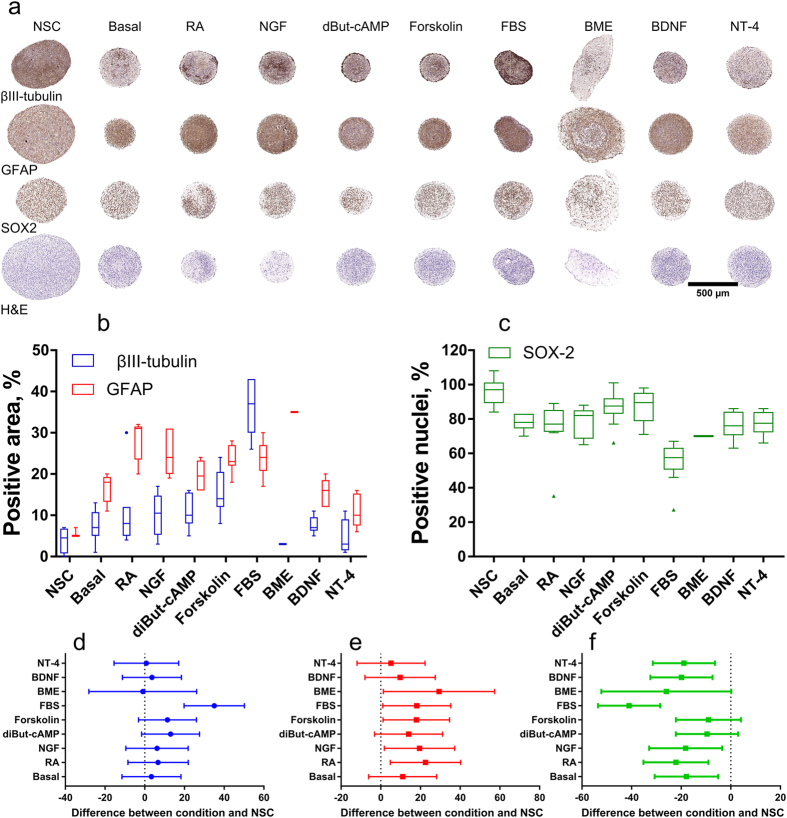
Neural stem cell differentiation in different media compositions. (**a**) Scanned images of neurospheres stained for markers of neuronal (βIII-tubulin) and glial (GFAP) differentiation, progenitors (SOX-2) and morphology (H&E). Different media conditions are arranged in columns, while the markers are arranged in rows. Scale bar 500 μm. (**b**) Tukey boxplot of the area positive for GFAP (red, left box) and βIII-tubulin (blue, right box) compared to the total area of the sphere. (**c**) Tukey boxplot of the number of nuclei positive for SOX-2 compared to the total number of nuclei in the spheroid. For (**b**) and (**c**) the line represents the median percentage, the box is formed by the first and third quartile, whiskers are 1.5 times the quartiles and points represent outliers. (**d**) to (**f**)- Graphs of the magnitude of the difference between the various differentiation media and NSC medium for βIII-tubulin (**d**), GFAP (**e**) and SOX-2(**f**). Dots (**d**) and squares (**e**,**f**) represent the mean difference, while the error bars are 95%CIs from the ANOVA analysis with Dunnet’s multiple comparison test follow-up.

Spheroid tissue microarrays were used to rapidly screen ten different media compositions (Table 1 in Methods) for their ability to promote neural differentiation in human fetal brain neurospheres (Fig. 5). After 14 days of *in vitro* culture, the neurospheres were embedded in microarrays, sectioned and stained for progenitors (SOX-2), neuronal (βIII-tubulin) or glial (GFAP) differentiation (Fig. 5a). The average size of the neurosphere sections from spheroids cultured in stem cell media was 600–800 μm, while the spheres in differentiation conditions were 300–400 μm in diameter. The images show little differentiation and high number of SOX-2 positive cells in neural stem cell (NSC) media, while neuronal and glial differentiation was visible in all other conditions. Retinoic acid induced differentiation in clusters within the spheroid, creating patches of neural networks in the neurospheres. Neurospheres in Basal media, media rich in NGF, BDNF and NT-4 showed differentiation in the core of the spheroid. In addition to the differentiation seen in the core, diBut-cAMP and forskolin also resulted in pronounced neuronal differentiation in the periphery of the spheroids. The addition of Basement membrane extract induced outgrowth from the neurospheres into an extracellular-matrix rich periphery and mainly glial differentiation. The addition of fetal bovine serum induced the most differentiation toward neuronal and glial lineages and stood out from all other conditions.

The visually observed patterns in differentiation from the spheroid microarrays were confirmed after automated image analysis (Supplementary macros 1 and 2). The percent of the spheroid area stained for βIII-tubulin or GFAP (Fig. 5b) and the relative proportion of SOX-2 positive nuclei (Fig. 5c) in all conditions were compared to NSC media. Neurospheres cultured in the presence of EGF and FGF showed around 5% differentiation to neurons and glia and 96% of nuclei were positive for SOX-2. The only condition which yielded a statistically significant increase in neural differentiation was the addition of serum as determined by Analysis of Variance (ANOVA) analysis with Dunnet’s correction for multiple comparisons for βIII-tubulin (Fig. 5d). In this condition, 39% of the spheroid area was positive for βIII-tubulin, which is a difference of 35% compared to NSC neurospheres (95% CI was 23% to 47%, p = 0.0001). The small magnitude of the neuronal differentiation in the other media compositions (median difference with NSC medium of 6%) resulted in no statistical difference for these conditions. In contrast, the same analysis for glial differentiation showed statistically significant differences compared to NSC medium for RA, NGF, Forskolin, FBS and BME (Fig. 5e). The median difference for all conditions was 18% more glial differentiation and the highest difference was seen in BME (35% positive) and retinoic acid (28% positive) compared to 6% GFAP positive cells in NSC medium. The increase in neurons and astrocytes in all differentiation conditions was underpinned by a drop in the number of cells positive for the progenitor marker SOX-2 (Fig. 5c). The number of progenitors decreased to 55% in serum and to around 80% the other differentiation conditions compared to 96% in NSC medium.

### Applications in drug screening

**Figure 6:**
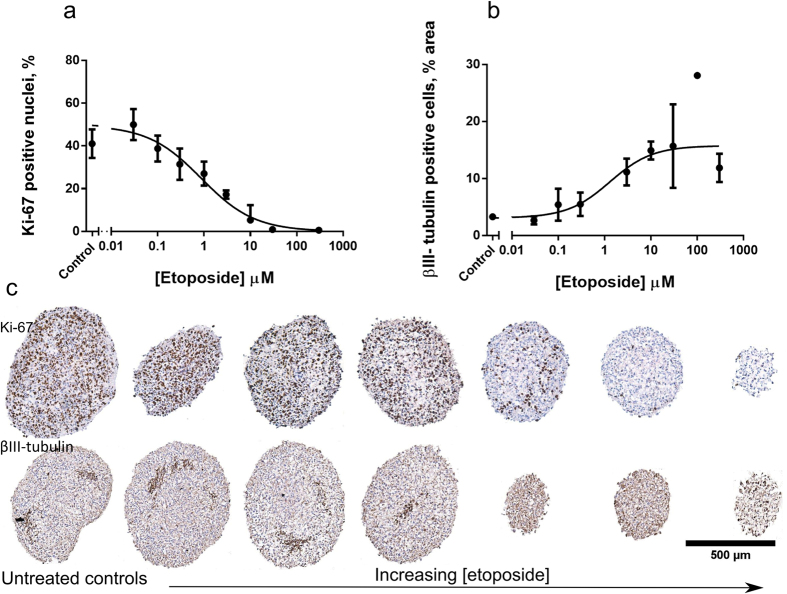
Effects of etoposide on the proliferation and neuronal differentiation of fetal brain neurospheres. (**a**) Ratio of Ki-67 positive nuclei (in percent) as a function of increasing etoposide concentration. (**b**) Relative area positive for βIII tubulin (%). Dots are mean values derived from 3 sections from 3 experimental repeats, error bars are SDs. Four parameter-dose response curves were fitted to the data. (**c**) Representative images of untreated spheroids (left) and spheroids at increasing concentrations of etoposide stained for Ki-67(top) and βIII-tubulin (bottom). Scale bar 500 μm.

Spheroid microarrays were used to quantify the effect of chemotherapy on human normal brain neurospheres by staining for proliferation (Ki-67) and neuronal markers (βIII-tubulin) (Fig. 6). Treatment with increasing concentrations of etoposide induced a dose-dependent drop in proliferation (Fig. 6a). This was accompanied by an increase in differentiation (Fig. 6b) at levels consistent with a reduction of spheroid volume and decrease in metabolic activity^10^. While untreated neurospheres had around 50% proliferating cells, etoposide treatment stopped proliferation at concentrations above 10 μM (IC50 = 0.9 μM 95%CI 0.6 to 1.3 μM). The reduction of proliferation was followed by an increase in the relative percentage of βIII-tubulin cells from 3% to over 16% area positive for the neuronal marker (IC50 = 1.3 μM, 95%CI 0.2–4.9 μM). In contrast to the healthy neurons with multiple interconnected processes in the control spheroids, the βIII-tubulin expressing cells at high etoposide concentrations (>30 μM) were rounded with apoptotic bodies and blebbing (Fig. 6c).

## Discussion

The spheroid microarray technology we have developed arranges spheroids into a single array before downstream processing, greatly simplifying the simultaneous analysis of many spheroids. This expands the applicability domain of cell line and primary tissue microarrays derived from *in vitro* cultures. In oncology, the technology can be used to monitor drug-dependent biomarker expression and identify markers of resistance. In the field of companion diagnostics, applications include controlling the sensitivity of antibody staining in quantifying drug-target expression. In the fields of stem cells and tissue engineering, this technology can monitor microtissue organization and differentiation patterns. For toxicological purposes, spheroid microarrays can be used to identify changes in gene expression associated with adverse outcome pathways of toxicity at non-cytotoxic concentrations.

This is the first device which allows building tissue microarrays of spheroids cultured under different conditions, or from different cell-lines, straight from the culture plate. It is compatible with all culture systems, but is best suited to spheroids grown in high-throughput plate-based formats. Cell-line specimens have been employed as on-slide quality controls for tissue microarrays^17^,^18^, because of their stable^19^ and precisely-controlled^20^ protein expression. However, the methods so far have involved separate processing of each culture and microarray assembly from multiple blocks^17^,^21^. The slow and cumbersome nature of this process has confined the use of cell-lines to quality control applications, whilst our approach considerably broadens their potential applications.

Arraying the spheroids straight from the culture plate, using a preformed mold, before paraffin infiltration saves up to 65 histology cassettes per experimental run, valuable researcher time and space in the embedding equipment while preventing mislabeling. Although we used paraffin embedding in the examples shown here, the system could also be applied for embedding spheroids for cryosectioning. Embedding the microtissues at this stage is advantageous because the spheroids are easy to distinguish from the transparent buffer solutions, can be picked up with a pipette and positioned in the array.

While there are other possible ways of making spheroid microarrays, they are immensely more labor-intensive. The spheroids can be embedded in individual paraffin or cryomolds, then a manual or automatic microarrayer can be used to arrange tissue from each donor mold into one recipient acceptor array. Although this process is commonly applied to macroscopic samples of patient tissue, it is less suited for spheroids. Using the latter methodology would result in large numbers of donor blocks, would require complicated positioning to pinpoint the small (0.5–1 mm) spheroid tissues and would take a considerable amount of time.

Our device has a one-piece design, which eliminates the need to keep constant pressure and frees up researchers’ hands. This is in contrast with a device described in a recent patent application, WO2015069742^22^, which uses a two-part device, requiring external pressure to keep a constant seal. Another unique feature of our device is that the resultant agarose molds can be inspected for defects before spheroid addition. Moreover the bottom surface of the array has a thin layer of agarose, allowing researchers to evenly trim the surface of the final paraffin block before reaching the spheroids during cutting. Finally, it is easier to separate the hard plastic pegs of this device from the agarose gel, compared to separating soft agarose pegs from a rigid mold^22^. This feature essentially eliminates spheroid loss and mold separation problems.

To illustrate the broad applicability of the technology, we have demonstrated its utility for examining morphology using spheroids derived from different cell-lines; staining for high, medium and low level expression markers by immunohistochemistry; investigating differentiation of neural stem cells under different culture conditions; and assessing cytotoxicity/neurotoxicity in normal fetal brain cell cultures.

Some of the cell-lines here formed loose aggregates (OVCAR3), irregularly shaped structures (HCC1806) and spheroids with a necrotic core (MDA-MB-361, MCF7). In contrast, the cell-lines used in our previous publications formed round spheroids with uniform density in their periphery and core, allowing spheroid volume to be used as a surrogate measure of spheroid viability in conjunction with metabolism measurements^10^,^11^. In the cases illustrated in this paper, fitting a single view spheroid area to a normal sphere may not be the most appropriate approach. The three-dimensional structure of the spheroids was revealed through sectioning, where necrotic cores or squashed and disk-like shapes became apparent. The ability of different cell-lines to form spheroids with varying morphology could be used as a means of authentication in addition to protein expression profiling and short tandem repeat-based authentication.

We went on to show that, in addition to being able to examine morphology, spheroid microarrays can be used to detect markers expressed in most cells, in approximately half of the cells or in a minor population of cells within spheroids (Fig. 3). While in this example, we used immunohistochemistry to detect protein markers, the methods could equally be applied to detect RNA or DNA markers e.g. using *in situ* hybridization methods. Marker expression can not only be quantified, but also localized within the spheroids. For markers expressed in a high proportion of cells within the spheroid (e.g. SOX-2 in our example) compared to proteins expressed only in a small number of cells, it would be possible to detect smaller relative changes in expression due to the low coefficient of variation. However, the main benefit of this system is that the spatial distribution of a biomarker expressed in a small population can be visualized. For example, in the case of βIII-tubulin, where neuronal cells were less than 5% of spheroid area, the patterns of expression and clustering were easily identified and quantified (Fig. 4c,d). In contrast, techniques which require spheroid disaggregation such as flow cytometry or lysis (e.g. blotting, gene expression arrays) would not be able to provide spatial information and detect heterogeneous biomarker expression in tissue samples respectively. This is a major advantage for experimental setups exploring patterning in three-dimensional structures, such as differentiation, development and tumor heterogeneity.

Taking this a stage further, the utility of spheroid microarrays in three-dimensional cell culture models of differentiation was illustrated by screening the ability of ten different media to induce differentiation in neural progenitors. Imaging the spheroids allows discrimination between substances causing neuronal patterning as clusters in the spheroid mass (Retinoic acid) and in the spheroid periphery (diBut-cAMP and Forskolin).

Lastly, we have shown that spheroid microarrays can also be used in drug screens to detect changes in proliferation and differentiation markers. For example, we have demonstrated the ability of cytotoxic chemotherapy (etoposide) to inhibit proliferation and induce differentiation in normal fetal brain cultures (Fig. 5). The IC50 values for Ki-67 and βIII-tubulin exhibited good correlation between each other and with our previously reported data for neurosphere volume and metabolic activity^10^. Apart from the ability to quantify these markers, imaging the spheroids revealed a change in their morphology at high etoposide concentrations indicating severe toxicity.

The technology can be improved by achieving a tighter control over spheroid distribution on the vertical axis and automating the transfer of spheroids from the culture plate to the arrays. Embedding the spheroids closer together on the vertical axis would enable the analysis of spheroids smaller than 300 μm. Automating spheroid transfer can be accomplished with a liquid-handling robot by programming the coordinates of donor plate wells and the receiver agarose wells. We hope that this open access manuscript and the freely available Mold-maker design would stimulate other researchers to develop the technique further.

We present a device which allows users to arrange up to 66 spheroids in a single plane for high-throughput biomarker analysis of three-dimensional cell cultures. This technology markedly improves the throughput possible in preclinical drug development using spheroid models. Use of such models has the potential to provide more clinically-relevant assessment of drug efficacy and safety than 2D *in vitro* data and can be used to replace and reduce the use of animals. We share the design files for the Mold-maker (Supplementary design 1) to facilitate the adoption of spheroid arrays and empower researchers to rapidly analyze the morphology, spatial patterns and biomarker expression in three-dimensional cell cultures. This will facilitate the rapid application of this technology by a wide spectrum of researchers working on cancer, stem cells and toxicology.

## Methods

### Design and rapid prototyping of the Mold-maker

The Mold-maker was designed in the Tinkercad 3D CAD design application. It was printed with a selective laser sintering printer (EOS Formiga P100) out of PA2200 (polyamide-12 powder) in the University of Nottingham Additive Manufacturing and 3D Printing Research Group.

### Embedding spheroids in microarray molds

The Mold-maker was spray-coated with a silicone mold release spray and left to dry (30 sec). Hot (50–70 °C) Type IA agarose solution (2% wt/vol in deionized water, 2 mL) was dispensed in a pre-warmed (37–50 °C) stainless steel histology base mold (25 × 20 mm). The Mold-maker was placed on top of the warm agarose solution and the base-mold gently pressed and tapped to remove any potential air bubbles trapped underneath the Mold-maker. The agarose solution was left to gel at room temperature (2 min, 21 °C) and subsequently the mold was transferred to a laboratory freezer and placed on a level surface (1 min, −18 °C). The Mold-maker was removed leaving an agarose mold of 66 wells. The previously-fixed spheroids (4% wt/vol paraformaldehyde solution in PBS, 16–24 h at 2–8 °C) were taken up with fixation media (7–8 μL) from each well using a 20 μL pipette tip with the top section cut-off to facilitate spheroid collection. After each well was filled with aqueous media from the spheroid plate (4% wt/vol paraformaldehyde solution in PBS), the whole mold was centrifuged (1 min, 100 g). The agarose molds were centrifuged by taping the stainless steel molds to the top of a 50 ml swing rotor centrifuge adaptor. The mold was quickly warmed (5 s on a hotplate) to reach 37–40 °C and low-gelling agarose (0.5 mL, 2% wt/vol in deionized water) was slowly dispensed on the side of the mold to seal the spheroids. The agarose array was left to gel (5 min at room temperature, 1 min at −18 °C), placed in a histological cassette and processed in a tissue processor overnight. The samples were dehydrated in a series of alcohol solutions with increasing concentrations (one bath of 50%, 70%, 90%, and 4 baths of 100% methanol, 1 h each), cleared in xylene (3 baths, 1 h each) and infiltrated with molten paraffin (2 baths, 2 hours each). The molds were then embedded in paraffin on the next day. A video of this protocol can be found on: https://figshare.com/s/0d3706f760854a44f603.

### Culturing human fetal brain cells

Human fetal brain cells were cultured as described by Ivanov *et al*.^10^.

### Differentiation experiments

Human fetal brain cells (15,000 cells/well) were seeded in ten different types of media (100 μL) (Table 1). All additives were already present in the cell media at seeding with the exception of basement membrane extract (2 μl per well from 6 mg/ml stock solution of Cultrex-BME) which was added 24 h after seeding in basal media (50 μl/per well). Fresh media (100 μL) was added to each condition on day 3 and from then onwards old media (100 μL) was replaced with fresh media (100 μL) every other day.

### Drug treatment

Human fetal neurospheres (7,500 cell/well) were cultured in ultra-low attachment plates (3 days) and treated (48 h) with 9 half-logarithmically spaced increasing concentrations of etoposide (0.01 μM to 300 μM). The drug-containing media was replaced with fresh media and the neurospheres were cultured for another 48 h without the drug as described by Ivanov *et al*.^10^. The spheroids were then imaged for volume analysis, treated with Resazurin to measure metabolism, fixed (4% wt/vol paraformaldehyde in PBS, 24 h, 2–8 °C) and embedded in microarray molds.

### Multi cell-line spheroid arrays

Cancer cell lines from breast (MCF7, MDA-MB-231, MDA-MB-361 and HCC1806), colon (HCT116, RKO), gastric (N87), brain (U87), pancreatic (BXPC3), ovarian cancer (OVCAR-III) and osteosarcoma (791T) were seeded at different cell densities (4, 8 and 16 × 10^3^ cells/well) in 100 μL of media (Table 2). The plates were centrifuged lightly (100 g, 3 min) when no BME was added, and more intensely (1000 g, 10 min) if 250 μg/mL of BME was added to encourage rapid spheroid formation. They were fed fresh media (100 μL) on day 2 and fixed on day 4 (4% PFA, 16–24 h) before embedding in spheroid microarrays.

### Uniformity of embedding depth experiments

The agarose molds were prepared as described above and magenta-colored acrylic spheres (radius around 500 μm) were placed into the agarose wells, centrifuged (100 g, 3 min) and covered with low-gelling agarose solution (0.5 mL, 2% wt/vol). The molds were cut along each row, placed on a level microscope slide and imaged on their side with a Canon PowerShot SX220HS camera mounted on a tripod. The images were calibrated with a millimeter sized ruler (1 pixel was equivalent to 25 μm) and the resolving power was determined to be 50–60 μm using the Fourier-transform function in ImageJ. Depth uniformity was analyzed in ImageJ. The images were cropped and rotated so that the microscope slide angle was adjusted to 0°. The images were then converted to 16-bit and thresholded (Default method) to separate the beads from background. After noise removal (Despeckle), applying Minimum (2 px) and Maximum (2 px) filters to remove small specks, all bead particles were analyzed using the (Analyze Particles function in ImageJ, area >10,000 μm^2^). The lower edge position on the Y-axis for each bead was determined by using the bounding triangle measurement in ImageJ. It was calculated by summing the value of the Y-coordinate of the upper-left corner of the bounding rectangle (BY) and its height. The median value for the lower edge was calculated for each of the six sections in three independent experiments and the differences were combined into one and plotted on a frequency plot.

### Immunohistochemistry

Spheroid microarray molds were cut (4 μm sections) and placed on poly-lysine coated slides to dry (24 h, 25 °C). Paraffin section areas were lined with a solvent resistant marker (Securline). Slides were dewaxed in 3 xylene baths (5 min each) and rehydrated in 2 methanol baths (1 min). Hematoxylin and eosin staining (H&E) was performed with Mayer’s Haemalum (3 min) and eosin (3 min).

For immunohistochemistry endogenous peroxidase activity was blocked in hydrogen peroxide (30 min, 1% in Methanol). Antigen retrieval was done by microwaving (30 min, 98 °C) in citrate buffer (10 mM, pH = 6). Blocking was done in serum (20% vol/vol in PBS) from the species matching the species of the secondary antibody. Primary antibody incubation (Table 3) was 1 h, 25 °C for all antibodies except for SOX-2 (2 h, 25 °C), followed by incubation with the secondary antibody (30 min, 25 °C).

Biotinylated secondary antibodies were followed by signal amplification (30 min) with ABC Complex (Vectastain, HRP). Diaminobenzidine (DAB) was used to visualize positive staining (3–7 min) and hematoxylin to counterstain (3 min). Slides were rehydrated in 3 methanol baths (1 min each) and cleared in and 2 xylene baths (3 min each) before cover-slipping with dibutyl phthalate in xylene (DPX).

### Imaging the spheroid microarrays

The IHC stained drug treatment spheroid arrays were imaged with a camera-equipped light microscope (Leica DM LB 30T and DFC-480) with the vendor-supplied software (Qwin 3.5.1). Uneven background illumination was corrected with an automated ImageJ macro code (Supplementary macro 3) based on the algorithm described by G. Landini^23^. Due to the low-throughput nature of the manual imaging setup all further images were automatically acquired with whole slide scanners.

The Hamamatsu Nanozoomer 2.0-HT imaged the H&E-stained mixed spheroid arrays, while the 3DHistech Pannoramic 250 Flash imaged the IHC signal uniformity and neural differentiation arrays. All images were acquired at 20x magnification, the Panoramic 250 images were acquired in extended focus mode with 7 focus levels and 5 μm step size. The spheroid images were exported from scans as separate images for each spheroid (JPEG-compressed tiffs from Panoramic.mrxs files or jpegs for the Nanozoomer.ndpi scans).

### IHC image analysis

Two custom written macros were used to analyze the background-corrected light microscope images or the exported scanned separate spheroid images in ImageJ. We have used the Fiji distribution of ImageJ with additional Image I/O and Jimi plugins to open compressed tiffs. Supplementary macro 1 works on cytoplasmic stains, while Supplementary macro 2 is optimized for nuclear stains. Both macros import the images for analysis from one images-only folder into an image stack. They run a minimum Z-projection to identify the smallest area containing all spheroids and allow the user to crop the extra white space. Afterwards the ImageJ Colour Deconvolution plug-in developed by G Landini based on the method of Ruifrok and Johnston^24^ was used to separate the Hematoxylin (H) and DAB staining in the images. For cytoplasmic stains, the images with the H component were then thresholded by the user to encompass the whole spheroid area. The DAB-component images were thresholded to recognize the DAB positive cells using appropriate positive and negative controls. The cytoplasmic macro then outputs a separate folder with the values of the thresholds, the total area of stained for H and DAB, and an image sequence of quality control images with green outlines for the recognized spheroid tissues and magenta outlines for the positive areas. The percentage of positive cells for cytoplasmic stains (GFAP, βIII-tubulin) was derived by dividing the total area positive for DAB by the total area positive for H in each image. In the nuclear stain macro the user would threshold solely the nuclei and not the whole spheroid area. Moreover, the code was optimized to detect solely nuclei by employing Despeckle, Median (2 px), Minimum and Maximum (2 px) filters, Watershed segmentation, as well as a minimum nuclear size (defined in line 55). Macro output from the nuclear staining macro also includes the number of nuclei positive for H and DAB. In the analysis step the percentage positive cells for nuclear stains (Ki-67, SOX-2) was derived by dividing the number of DAB-positive nuclei by the number of H-positive nuclei.

### Statistical analysis

Data were analyzed in GraphPad Prism version 7. For the single plane experiments, the deviations from the median lower edge from three experiments were plotted on a histogram plot with 50 μm bins. In the signal uniformity experiments, the separation between negative control samples and samples with high, medium and low expression of protein biomarkers was assessed using the robust Z′ based on equation (1), with acceptance criteria of Z′ > 0.5^16^. The differences in the differentiation experiments were quantified and plotted with an ANOVA analysis employing Dunnet’s correction for multiple comparisons. Data for the percentage of Ki-67 and βIII-tubulin positive cells in the drug treatment experiments were fitted to a four-parameter sigmoidal dose-response curve. The bottom of the Ki-67 dose-response curve was constrained to zero, while the βIII-tubulin dose response curve was left unconstrained.

## Additional Information

**How to cite this article**: Ivanov, D. P. and Grabowska, A. M. Spheroid arrays for high-throughput single-cell analysis of spatial patterns and biomarker expression in 3D. *Sci. Rep.* **7**, 41160; doi: 10.1038/srep41160 (2017).

**Publisher's note:** Springer Nature remains neutral with regard to jurisdictional claims in published maps and institutional affiliations.

## Supplementary Material

Supplementary Information

## Figures and Tables

**Figure 1:**
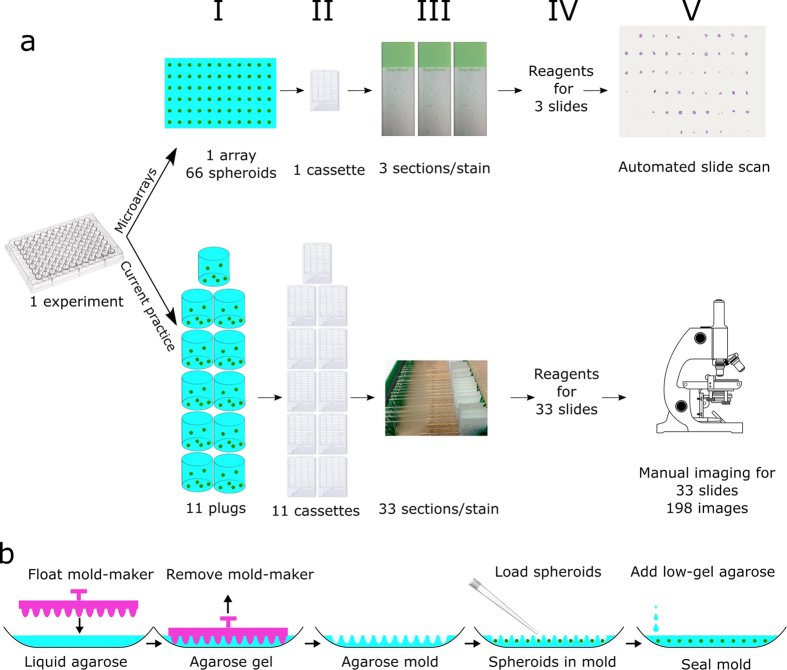
Spheroid microarray technology overview and mold making procedure. (**a**) The current workflow to analyze spheroid histology requires separate processing of spheroids representing different conditions and results in many samples which need to be embedded (I), processed (II), sectioned (III), stained(IV) and imaged (V) separately. The random distribution of spheroids in different planes requires manual imaging and further takes up researcher and equipment time. Embedding multiple conditions on the same array (top) reduces the number of samples 11 times resulting in economies in reagents and hands-on time as well as the possibility for automated imaging of all spheroids located in the same plane. (**b**) Spheroid microarrays are made by pouring liquid agarose solution in histology molds and floating the Mold-maker on top of the solution. Once the agarose cools down and gels, the Mold-maker is removed and the spheroids are loaded into the wells of the resulting agarose mold. The mold is sealed with low-gelling agarose and is processed for histology.

**Table 1:** Table of differentiation media composition.

Basal media (100 mL)	Additions	Short name
DMEM (47.5 mL)	EGF(20 ng/mL) and FGF(10 ng/mL)	*NSC*
F12 (47.5 mL)	—	*Basal*
B27 (2 mL)	Retinoic acid (300 ng/mL)	*RA*
N2 (1 mL)	NGF (10 ng/mL)	*NGF*
Heparin (5 μg/mL)	diBut-cAMP (500 μg/mL)	*diBut-cAMP*
	Forskolin (4.1 μg/mL)	*Forskolin*
	Fetal bovine Serum (5% vol/vol)	*Serum*/*FBS*
	Cultrex BME (250 μg/mL)	*BME*
	BDNF (50 ng/mL)	*BDNF*
	NT-4 (10 ng/mL)	*NT-4*

DMEM- Dulbecco’s Modified Eagle’s medium, high-glucose (4.5 g/L), sodium pyruvate (1 mM). F12-Ham’s F12 medium with HEPES (4-(2-hydroxyethyl)-1-piperazineethanesulfonic acid, 25 mM. B27-supplement without insulin (50X) (Life Technologies). N2 supplement (100X) (Life Technologies). Heparin sodium salt from porcine intestinal mucosa (Sigma Aldrich, Grade I-A, cell culture tested). EGF- Recombinant human epidermal growth factor (EGF, Life Technologies). FGF-recombinant human basic-Fibroblast growth factor (FGF, AA 10–155, Life Technologies). NGF-Nerve Growth Factor human recombinant (Sino Biological, Life Technologies). diBut-cAMP - N6,2′-O-Dibutyryladenosine 3′,5′-cyclic monophosphate sodium salt (Sigma-Andrich). Forskolin (Calbiochem). Cultrex Basement membrane extract (Trevigen). BDNF- Brain Derived Neurotrophic Factor (human recombinant, Source Bioscience), NT-4- Neurotrophic factor (human recombinant, Source Bioscience).

**Table 2:** Cell-lines used to build the mixed spheroid array.

Cell-line	Basal Media	Basement membrane extract addition (250 μg/mL)
OVCAR-IIII	RPMI + Insulin	+
HCT116	DMEM	−
HCC 1806	DMEM	+
MDA-MB-231	RPMI	+
MDA-MB-361	RPMI	+
MCF7	RPMI	−
RKO	DMEM	+
N87	RPMI	−
BXPC3	RPMI	−
U87	RPMI	−
791T	MEM	−

**Table 3:** List of antibodies (Ab) used in the IHC experiments.

Antibodies	Dilution	Sources	Cat. no
**Primary**			
Mouse monoclonal anti-Ki-67 Ab (MIB-1)	1:150	DAKO	M7240
Mouse monoclonal anti-βIII-tubulin Ab (TU-20)	1:500	Abcam	ab7751
Rabbit polyclonal anti-GFAP Ab	1:1000	Abcam	ab7260
Rabbit polyclonal anti-SOX-2 Ab	1:5000	Abcam	ab97959
**Secondary**			
Polyclonal rabbit anti-mouse HRP	1:300	DAKO	P0161
Polyclonal swine anti-rabbit biotinylated	1:300	DAKO	E0353
