# Supplementary material for: Spheroid arrays for high-throughput single-cell analysis of spatial patterns and biomarker expression in 3D
Source: Sci Rep. 2017 Jan 30;7:41160. doi: 10.1038/srep41160 (PMC5278392; doi:10.1038/srep41160)
Supplement: Supplementary Information [file srep41160-s1.pdf]

Spheroid arrays for high-throughput single-cell analysis of spatial patterns and biomarker expression in 3D

Authors:

***Delyan P. Ivanov[a], Anna M. Grabowska[a]**

a- Cancer Biology, Division of Cancer and Stem Cells, School of Medicine, Queen's Medical Centre, University of Nottingham, Nottingham, UK

***Correspondence to :** delyan.ivanov@nottingham.ac.uk, Delyan Ivanov, Cancer Biology, Division of Cancer and Stem Cells, Queen's Medical Centre, University of Nottingham, Nottingham NG7 2UH, UK

Telephone: +44 (0) 115 8231135

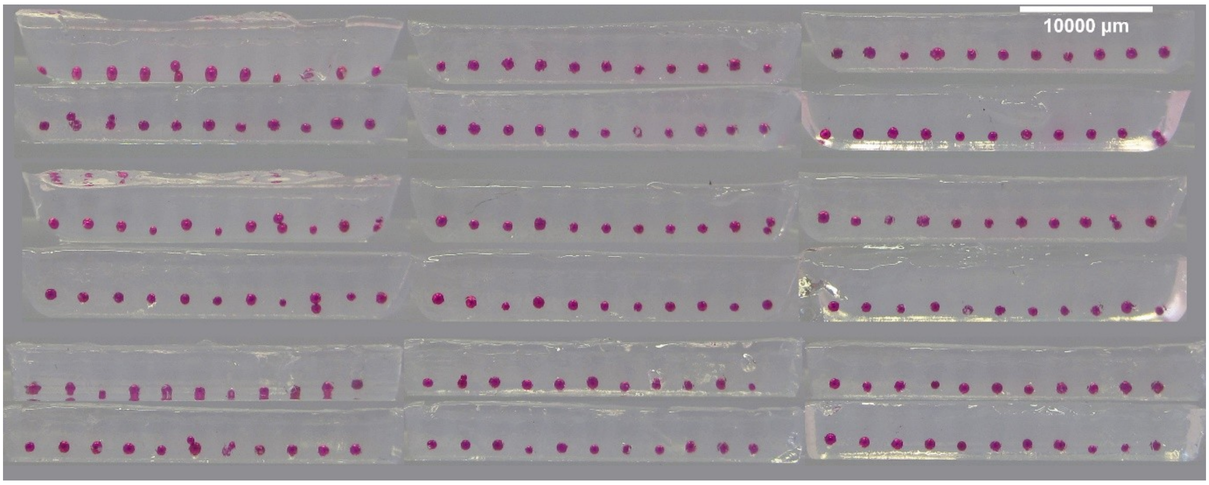

Supplementary Figure 1. Cross-section photographs of three microarray molds. *Magenta-colored plastic beads (r=500µm) were embedded in three separate arrays. The arrays were sectioned along each row, photographed and combined in a single image.*

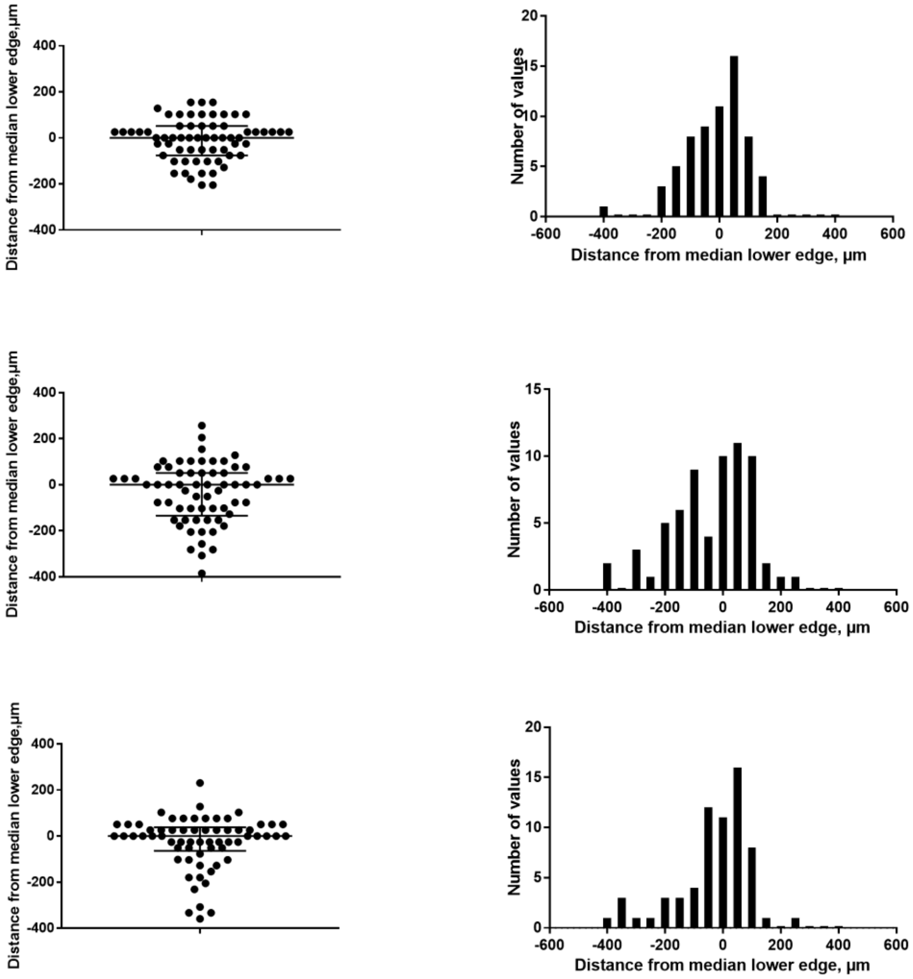

Supplementary Figure 2. Uniformity of embedding depth for three arrays*. Left panels- Distribution of the distance from the median lower edge for each bead from three independent experiments. Line is median, bars represent the interquartile range. Right panels- frequency plot of distance from the median lower edge for the corresponding experiments.*

The Supplementary Design file to print the arrays, Supplementary macro 1 for cytoplasmic images, Supplementary macro 2 for nuclear stains and Supplementary macro 3 to correct uneven background illumination are available to download from the Figshare open database.

Supplementary design file DOI: 10.6084/m9.figshare.4269173

Supplementary macro 1 DOI: 10.6084/m9.figshare.4269194

Supplementary macro 2 DOI: 10.6084/m9.figshare.4269191

Supplementary macro 3 DOI: 10.6084/m9.figshare.4269185
